# Preoperative blood-brain barrier disruption increased risk of postoperative cognitive decline after coronary artery bypass grafting: a prospective cohort study

**DOI:** 10.1097/JS9.0000000000002902

**Published:** 2025-07-08

**Authors:** Weijing Wang, Sen Zhang, Lu Yu, Tianjie Wang, Guanxi Wang, Fei Xu, Shujuan Li

**Affiliations:** a Department of Neurology, Fuwai Hospital, National Center for Cardiovascular Diseases, Chinese Academy of Medical Sciences and Peking Union Medical College, Beijing, China; b Department of Adult Cardiac Center, Fuwai Hospital, National Center for Cardiovascular Diseases, Chinese Academy of Medical Sciences and Peking Union Medical College, Beijing, China; c Department of Radiology, Fuwai Hospital, National Center for Cardiovascular Diseases, Chinese Academy of Medical Sciences and Peking Union Medical College, Beijing, China; d Department of Cardiology, Fuwai Hospital, National Center for Cardiovascular Diseases, Chinese Academy of Medical Sciences and Peking Union Medical College, Beijing, China

**Keywords:** cognitive dysfunction, coronary artery bypass grafting (CABG), coronary artery disease (CAD), neuropsychological tests, postoperative cognitive complications

## Abstract

**Background:**

Cognitive decline after coronary artery bypass grafting (CABG) surgery is common and significantly impacts the long-term prognosis of coronary artery disease (CAD) patients. Nevertheless, the mechanisms of postoperative cognitive decline (POCD) and its early neuroimaging indicators remain unclear.

**Objectives:**

To explore whether blood-brain barrier disruption of computed tomography perfusion (CTP) is associated with POCD in patients with CABG.

**Methods:**

The study involved 116 participants (mean age 63.85 ± 8.10 years) with CAD who underwent CABG surgery as part of the IACV Study. A CTP scan was performed before the surgery to assess the permeable surface (PS) values of the frontal and temporal lobes. The Montreal Cognitive Assessment (MoCA) neurological scale was used to measure cognitive function before the surgery and again before discharge.

**Results:**

Overall, 24 (20.69%) developed POCD. All patients underwent the same anesthesia and postoperative analgesia, with no difference in postoperative blood loss and days of hospital discharge between the two groups. Patients with POCD exhibited higher PS in the right frontal lobe (0.20 [0.10, 0.30] vs 0.13 [0.07, 0.18], *P* = 0.009) and left frontal lobe (0.23 [0.11, 0.28] vs 0.14 [0.08, 0.22], *P* = 0.030). A notable decrease in total MoCA scores from 20.7 to 14.0 was seen in patients with POCD. Each 0.05 ml/100 g/min increase in PS of the right frontal lobe was associated with 1.32 times increase in the risk of developing POCD. Higher baseline PS values correlated with a greater decline in MoCA scores post-surgery (*ρ* = −0.231, *P* = 0.0214).

**Conclusions:**

Almost one-fifth of patients develop POCD following CABG surgery, and preoperative elevation of frontal lobe PS is associated with POCD. These findings suggest that CTP-PS may serve as an imaging biomarker to identify patients at high risk for POCD.

## Introduction

Postoperative cognitive decline (POCD) is the most common brain injury after coronary artery bypass grafting (CABG) and significantly affects patients’ quality of life and long-term prognosis^[1]^. Studies indicate that cognitive decline occurs in 53% of patients at discharge, 36% at 6 weeks, 24% at 6 months, and 42% at 5 years, with cognitive function at discharge being a significant predictor of long-term outcomes^[2]^. These outcomes affirm the high incidence and duration of cognitive deterioration following CABG surgery, indicating a trend of initial enhancement succeeded by a later deterioration, which is linked to the existence of cognitive decline in the early postoperative period.

Extensive research has highlighted the prevalence of POCD among CABG patients^[2]^, emphasizing the need to understand its potential to cause long-term cognitive decline and the importance of developing proactive intervention strategies^[2–4]^. MR imaging shows that blood-brain barrier (BBB) disruption occurs post-cardiac surgery in nearly half of the patients studied, particularly in those who received gadolinium within 24 hours of surgery^[3]^. However, this was a small-scale study focusing only on postoperative BBB function without linking it to preoperative conditions or POCD. Recent study by Glumac *et al*^[5]^ showed that the inflammatory response plays an important role in POCD development. Moreover, it seems that inflammatory response further causing perioperative disruption of BBB.

POCD is a common neurological complication after CABG surgery, which affects the long-term prognosis of patients. BBB disruption is considered one of the possible causes of POCD. Therefore, preoperative measurement of permeable surface (PS) values of computed tomography perfusion (CTP) is used to quantify BBB damage and study the relationship between preoperative BBB destruction and postoperative POCD. It is hoped that this study can find risk predictors for POCD after CABG.

## Methods

### Study design and protocol

**Figure 1.**
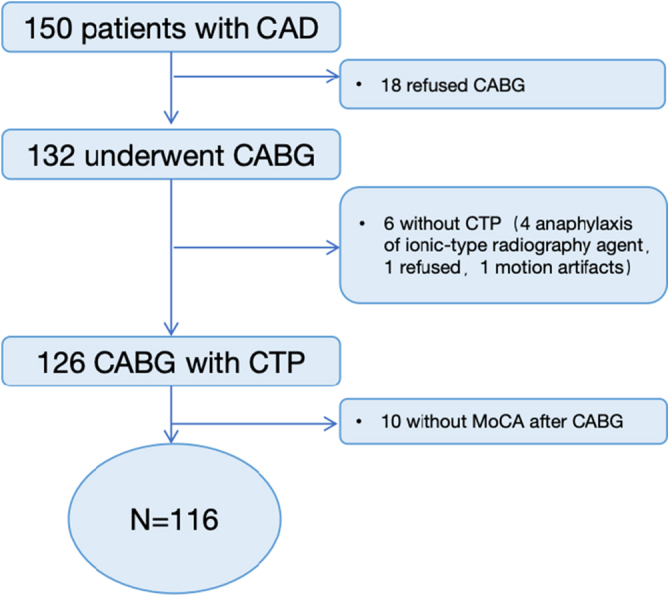
Flowchart.

This is a single-center prospective cohort study conducted at a single university teaching hospital. Between February and December 2023, a total of 150 patients were initially screened (Fig. 1). Of these, 132 underwent CABG. Pre-surgery neurocognitive evaluations were completed for 132 patients, forming the study’s final analysis group. Out of them, 126 underwent CTP. Ten patients declined postoperative neurocognitive evaluations. Ultimately, 116 patients were enrolled in this prospective, observational study examining cognitive impairment following CABG surgery. The inclusion criteria were: age ≥18 years; surgical indications for CABG following the 2021 ACC/AHA/SCAI Coronary Artery Revascularization Guidelines; informed consent obtained. The exclusion criteria included: history of cranial surgery; allergies to iodinated contrast agents; poor image quality precluding post-processing analysis; mental illness or severe baseline cognitive impairment (MoCA <16 points); estimated survival of less than 6 months due to major organ diseases; refusal to participate.

**HIGHLIGHTS**

- In this prospective study, computed tomography perfusion was employed to assess blood-brain barrier (BBB) function before coronary artery bypass grafting (CABG) surgery, aiming to uncover the correlation between baseline BBB condition and postoperative cognitive decline (POCD).
- This marks the first integration of neurostructural imaging with functional assessment to investigate neurocognitive changes in coronary artery disease (CAD) patients following CABG.
- Patients with CAD exhibit a higher susceptibility to cognitive decline.
- The preoperative elevation of PS may be due to BBB impairment, which is associated with POCD following CABG surgery.
- This study introduces a precise imaging technique for the early identification of individuals at risk for developing POCD before undergoing CABG surgery. Early detection may enable future studies to mitigate this risk.

After obtaining informed consent, patients underwent cognitive function evaluation using the MoCA scale and neuroimaging assessment with head computed tomography angiography + CTP. Subsequently, CABG surgery was performed. The neurologists conducting the evaluations were blinded to the imaging results. The studies involving human participants were reviewed and approved by the Ethics Committee of xx Hospital. The patients/participants provided their written informed consent to participate in this study. The work has been reported in line with the STROCSS criteria^[6]^.

### Neurocognitive assessment

Patients underwent a neurocognitive assessment to characterize their baseline cognitive functions before coronary artery bypass graft (CABG) surgery and prior to discharge (5–7 days after surgery). This assessment was conducted by a neurologist (XX) using the Montreal Cognitive Assessment (MoCA)^[7,8]^ to evaluate global cognition, attention, executive function, episodic memory, and verbal fluency. We believe that a 20% reduction in POCD score can be considered POCD, attributed to previous studies^[2,9]^.

Surgery, CPB, anesthesia management, and image acquisition are described in detail in the Supplemental Digital Content, available at: http://links.lww.com/JS9/E622.

### Image analysis

Permeable surface were calculated using the AW 4.6 workstation based on a deconvolution algorithm. The process was semi-automated with routine clinical practice settings. Automatic motion correction was employed to eliminate artifacts, and noise was further reduced using an automatic 4-D noise reduction tool. Inputs were taken from the cavernous segment of the internal carotid artery, and outputs were set to the superior sagittal sinus. Regions of interest (ROIs) around the frontal and temporal lobes were delineated bilaterally to determine perfusion values. A board-certified radiologist with over 5 years of experience in neuroradiology, blinded to clinical test results, performed the postprocessing.

### Data collection

All demographic data, baseline clinical characteristics, imaging tests (including CTA and CTP), and clinical neurocognitive assessments were prospectively and confidentially recorded in a database, maintaining researcher blinding.

### Statistical analysis

Descriptive statistics segmented the population based on POCD, indicated by a MoCA score at least 20% lower after CABG than before. Comparisons between patients with and without POCD used χ^2^ or Fisher’s exact tests for categorical variables, and Student’s *t*-test or Wilcoxon’s signed-rank test for continuous variables. Linear regression analyzed the relationship between right frontal lobe perfusion scores (PS) and baseline MoCA, including changes pre- and post-surgery. Logistic regression, with no adjusted variables, used POCD as the dependent variable and PS of the right frontal lobe as the independent variable, with results presented in a forest plot showing odds ratios (OR) and 95% confidence intervals. Tests were performed with GraphPad Prism (version 9.0, GraphPad Software, La Jolla, California) and R (R version 4.0.4, platform x 86_64-apple-darwin17.0).

## Results

### Clinical characteristics

From February to December 2023, a study involving 116 patients diagnosed with coronary artery disease (CAD) requiring CABG surgery was conducted. The average age of the participants was 63.9 years, with 92 of them being male (79.31%). Table 1 details cardiovascular risk factors, baseline comorbidities, and treatments. Of these patients, 24 (20.7%) patients experienced POCD, and 24 (20.7%) patients had a history of myocardial infarction. The MoCA score before CABG was 21.6. Notably, 98 patients (84.48%) showed neurocognitive decline before undergoing CABG, and 53 patients (45.69%) had cerebrovascular stenosis. Patients with POCD exhibited higher permeability surface (PS) in the right frontal lobe (0.20 [0.10, 0.30] vs 0.13 [0.07, 0.18], *P* = 0.009) and left frontal lobe (0.23 [0.11, 0.28] vs 0.14 [0.08, 0.22], *P* = 0.030).

**Table 1:** Baseline characteristics of the population

	Total (*n* = 116)	Without POCD (*n* = 92)	With POCD (*n* = 24)	*P*-value
AGE (y)	63.85 (±8.10)	63.60 (±8.35)	64.83 (±7.13)	0.62
Gender (male, %)	92 (79.31%)	73 (79.35%)	19 (79.17%)	1
BMI	25.44 (±2.58)	25.60 (±2.55)	24.86 (±2.68)	0.26
Previous MI	24 (20.69%)	19 (20.65%)	5 (20.83%)	1
Diabetes	57 (49.14%)	44 (47.83%)	13 (54.17%)	0.65
Hypertension	79 (68.10%)	60 (65.22%)	19 (79.17%)	0.23
Dyslipidemia	79 (68.10%)	63 (68.48%)	16 (66.67%)	1
Smoking				1
Never	58 (50.00%)	46 (50.00%)	12 (50.00%)	
Active	57 (49.14%)	45 (48.91%)	12 (50.00%)	
Former	1 (0.86%)	1 (1.09%)	0 (0.00%)	
LVEF (%)	59.34 (±9.39)	59.97 (±9.07)	56.92 (±10.36)	0.17
NT-pro-BNP (pg/ml)	212.00 [73.95, 556.00]	202.00 [73.95, 545.00]	217.00 [90.12, 843.50]	0.474
Crea (ummol/L)	69.35 [57.52, 81.95]	69.20 [56.43, 80.40]	70.00 [60.78, 83.75]	0.555
LDL (mmol/L)	2.36 (±0.96)	2.30 (±0.90)	2.61 (±1.15)	0.27
HbA1c (%)	6.96 (±2.24)	6.83 (±2.35)	7.46 (±1.74)	0.25
Aspirin	106 (91.38%)	84 (91.30%)	22 (91.67%)	1
Clopidogrel	74 (63.79%)	58 (63.04%)	16 (66.67%)	0.82
Ticagrelor	22 (18.97%)	18 (19.57%)	4 (16.67%)	1
Anticoagulants	41 (35.34%)	35 (38.04%)	6 (25.00%)	0.34
Statins	111 (95.69%)	88 (95.65%)	23 (95.83%)	1
pre_NCD	98 (84.48%)	74 (80.43%)	24 (100.00%)	0.19
CVD				0.87
Without CVD	63 (54.31%)	48 (52.17%)	15 (62.50%)	
Extracranial vascular stenosis	17 (14.66%)	14 (15.22%)	3 (12.50%)	
Intracranial vascular stenosis	28 (24.14%)	23 (25.00%)	5 (20.83%)	
Intracranial and extracranial vascular stenosis	8 (6.90%)	7 (7.61%)	1 (4.17%)	
Right frontal lobe	0.14 [0.08, 0.22]	0.13 [0.07, 0.18]	0.20 [0.10, 0.30]	0.009
Left frontal lobe	0.15 [0.08, 0.23]	0.14 [0.08, 0.22]	0.23 [0.11, 0.28]	0.030
Right temporal lobe	0.15 [0.09, 0.25]	0.14 [0.08, 0.24]	0.17 [0.09, 0.26]	0.567
Left temporal lobe	0.16 [0.08, 0.27]	0.16 [0.08, 0.25]	0.15 [0.08, 0.29]	0.867
MoCA before CABG	21.63 (±4.35)	21.87 (±4.41)	20.71 (±4.05)	0.19

Results are shown as mean (±standard deviation) or *n* (%).

CMD, cerebral microvascular dysfunction; CVD, cerebrovascular disease; LVEF, left ventricular ejection fraction; MI, myocardial infarction; MoCA, Montreal Cognitive Assessment; NCD, neurocognitive decline; POCD, postoperative cognitive decline; PS, permeability surface.

Table 2 presents the baseline characteristics of intraoperative and postoperative data. Thirty-two patients underwent off-pump CABG (27.59%), the average number of grafts was 4, and the average intensive care unit (ICU) stay was 23 hours. Patients with POCD had longer ICU stays 39.00 ([21.75, 108.25] hours vs 22.00 [17.00, 46.00] hours, *P* = 0.005). All patients underwent general anesthesia surgery, and postoperative pain treatment was routinely administered with oxycodone acetaminophen 5 mg per day for 3 days. Patients with and without POCD exhibit no significant difference in postoperative bleeding (569.0 ± 75.2 vs 572.8 ± 72.5, *P* = 0.828), nor is there a difference in the length of hospital stay (13.6 ± 3.9 vs 15.2 ± 5.9, *P* = 0.107).

**Table 2:** Baseline characteristics of intraprocedural data

	Total (*n* = 116)	Without POCD (*n* = 92)	With POCD (*n* = 24)	*P*-value
Off pump	32 (27.59%)	26 (28.26%)	6 (25.00%)	1
Number of grafts	4.00 [3.00, 4.00]	4.00 [3.00, 4.00]	3.50 [3.00, 4.00]	0.399
Duration of cardiopulmonary bypass	111.56 (±32.93)	110.61 (±32.17)	114.25 (±36.35)	0.747
Blood pressure	49.27 (±4.93)	49.48 (±5.06)	48.43 (±4.39)	0.365
Perfusion pressures	57.88 (±8.29)	57.95 (±8.01)	57.65 (±9.48)	0.880
Mechanical ventilation (h)	8.00 [6.00, 11.00]	7.00 [6.00, 10.00]	10.00 [6.00, 12.25]	0.121
ICU stay (h)	23.00 [17.00, 60.50]	22.00 [17.00, 46.00]	39.00 [21.75, 108.25]	0.005
Postoperative AMI	7 (9.09%)	5 (8.47%)	2 (11.11%)	0.660
Postoperative AKI	29 (37.66%)	23 (38.98%)	6 (33.33%)	0.780
Blood loss	569.8 (±13.9)	569.0 (±75.2)	572.8 (±72.5)	0.828
Hospital stay (h)	13.9 (±4.4)	13.6 (±3.9)	15.2 (±5.9)	0.107

Results are shown as mean (±standard deviation) or *n* (%).

AKI, acute kidney injury; AMI, acute myocardial infarction; POCD, postoperative cognitive decline.

### Neurocognitive assessment

**Figure 2.**
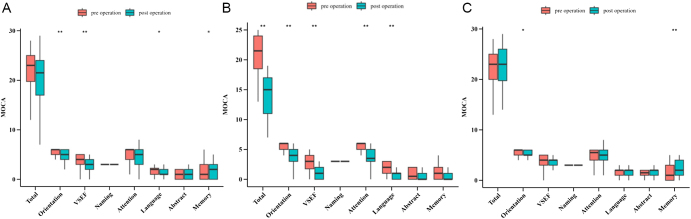
Neurocognitive assessment before and after the surgery in patients with and without POCD. (a) Pre- and post-operation MoCA for the overall population. (b) Pre- and post-operation MoCA for patients with POCD. (c) Pre- and post-operation MoCA for patients without POCD. VSEF, visual space and executive function; *P < 0.05; **P < 0.01.

Figure 2a illustrates significant reductions in neurocognitive assessment for the overall population. Patients with POCD showed declines in total MoCA score (20.7 vs 14.0), orientation (5.25 vs 3.63), visual-spatial and executive functions (2.80 vs 1.25), attention (5.17 vs 3.71), and language (1.88 vs 0.80) (Fig. 2b); patients without POCD showed no declines in total MoCA score (21.9 vs 22.1), visual-spatial and executive functions (3.73 vs 3.45), attention (4.69 vs 4.67), and language (1.82 vs 1.79) (Fig. 2c).

Draw a box plot with preoperative and postoperative grouping, where red represents preoperative cognitive indicators and blue represents postoperative cognitive indicators. The upper edge of the box represents the upper quartile, the lower edge represents the lower quartile, and the bold horizontal line in the middle represents the median. The Wilcoxon rank sum test results are marked at the top of the box plot. MoCA, Montreal Cognitive Assessment; VSEF, visual space and executive function; *P < 0.05; **P < 0.01.

### Brain CT perfusion

**Figure 3.**
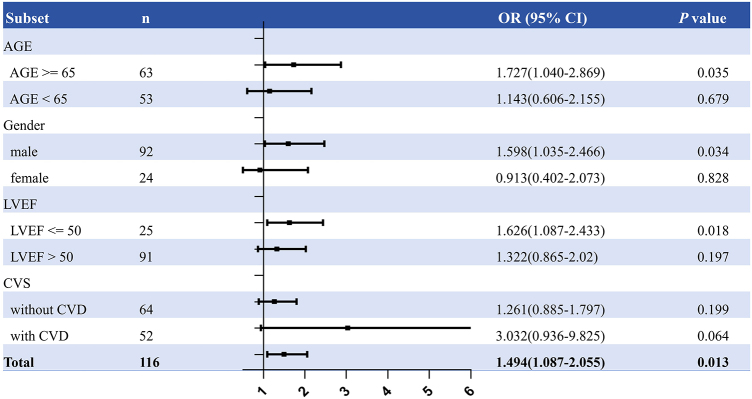
Relation between PS and postoperative cognitive decline.

**Figure 4.**
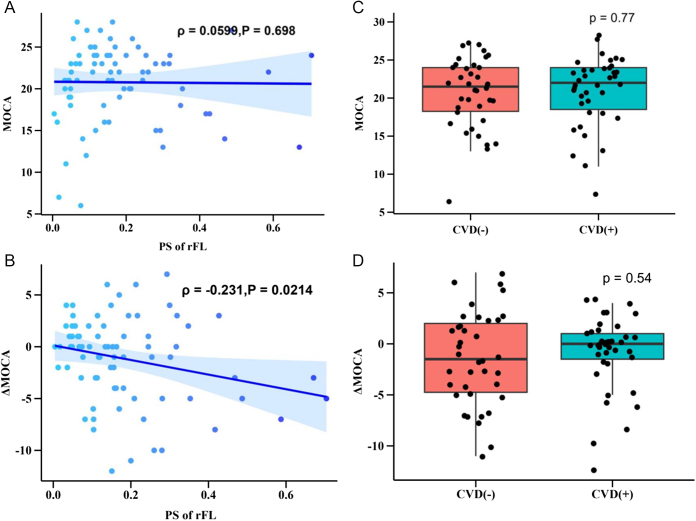
Relation between PS, CVD, and postoperative cognitive decline. (a) and (b) The relationship between PS of rFL (continuous variable) and baseline MoCA, as well as the difference in MoCA before and after surgery. Each point represents a sample, and linear regression (without adjusted variables) is used to visualize the correlation between the two, with the 95% confidence interval of the regression equation labeled (dark blue thick line and surrounding sky blue shadow). The upper right corner of the figure indicates the Spearman rank correlation coefficient and its one-sided test results for <0. (c) and (d) The distribution of baseline MoCA and changes in MoCA before and after surgery using CVD grouping. The *P*-value of Wilcoxon rank sum test is marked in the upper right corner. PS of rFL, PS of Right frontal lobe (ml/100 g/min); CVD, cerebrovascular disease; ΔMoCA, postoperative MoCA minus preoperative MoCA. (a) Relation between PS of rFL and baseline MoCA. (b) Relation between PS of rFL and ΔMoCA. (c) Relation between CVD and baseline MoCA. (d) Relation between CVD and ΔMoCA.

Figures 3 and 4 display findings related to PS and POCD. PS values were higher in patients with POCD compared to those without in both the right and left frontal lobes, as previously noted. This model suggests that each 0.05 increase in PS of the right frontal lobe increases the risk of POCD by a factor of 1.49, an association consistent across different age groups, genders, left ventricular ejection fractions, and cerebrovascular statuses (Fig. 3). Multivariate adjustments have been performed in the logistic regression models. Model 1 adjusted for gender and age, while Model 2 adjusted for gender, age, hypertension, diabetes, history of cerebrovascular disease, and baseline left ventricular ejection fraction (Table 3). Table 3 shows that elevated PS is an independent risk factor for POCD (OR 1.334 [1.113–1.599], *P* = 0.002).

For each 0.05 ml/100 g/min-increase in PS for the right lobe. Shown is a forest plot of the hazard ratio of POCD in prespecified subgroups. Using logistic regression with adjusted for age, gender, history of diabetes, history of hyperperfusion, LVEF, history of CVD variables, POCD as the dependent variable, and PS of rFL as the independent variable (for each 0.05 ml/100 g/min-increase in PS of rFL). PS of rFL, the permeability surface of the right frontal lobe; DM history, diabetes history; HT history, hypertension history; LVEF, left ventricular ejection fraction; CVS, cerebrovascular stenosis; CVD, cerebrovascular disease.

**Table 3:** Multivariable logistic regression analysis for POCD

	Unadjusted		
	Variable	OR (95% CI)	*P*-value
Unadjusted	rf PS	1.324 (1.112–1.577)	0.002
Model 1	Age	1.011 (0.952–1.075)	0.712
	Gender (male)	1.424 (0.405–5.003)	0.581
	rf PS	1.334 (1.113–1.599)	0.002
Model 2	Age	1.405 (0.380–5.202)	0.610
	Gender (male)	1.016 (0.953–1.084)	0.628
	Diabetes	1.220 (0.398–3.738)	0.727
	Hypertension	0.411 (0.115–1.467)	0.171
	LVEF (%)	0.967 (0.915–1.023)	0.242
	CVD	0.794 (0.283–2.229)	0.662
	rf PS	1.321 (1.086–1.605)	0.005

For each 0.05 ml/100 g/min-increase in rfPS.

ACP, antegrade cerebral perfusion; CI, confidence interval; LVEF, left ventricular ejection fraction; OR, odds ratio; rfPS, permeability surface for the right temporal lobe.

Model 1 adjusted for age, gender; Model 2 adjusted for age, gender, history of diabetes, history of hyperperfusion, LVEF, history of CVD.

Figure 4 indicates that there is no positive correlation between baseline PS and MoCA scores (*ρ* = 0.0144, *P* = 0.561). However, patients with higher baseline PS values are more likely to experience a significant decrease in MoCA scores post-surgery (*ρ* = −0.231, *P* = 0.0214). The analysis found no correlation between cerebrovascular stenosis and either baseline or postoperative MoCA scores (*P* = 0.77 and *P* = 0.54, respectively).

### Follow up

**Figure 5.**
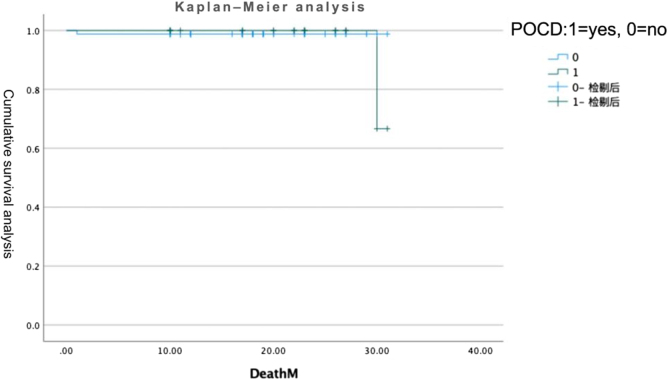
Kaplan–Meier analysis estimating overall survival between patients with POCD and without POCD (*P* = 0.768). POCD, postoperative cognitive dysfunction.

Long-term follow-up data have been supplemented in Figure 5. We conducted telephone follow-ups with all patients, among whom 102 completed the telephone follow-up, with 14 lost to follow-up (12%), and an average follow-up duration of 17 months. POCD is not associated with long-term mortality risk (*P* = 0.768).

## Discussion

**Figure 6.**
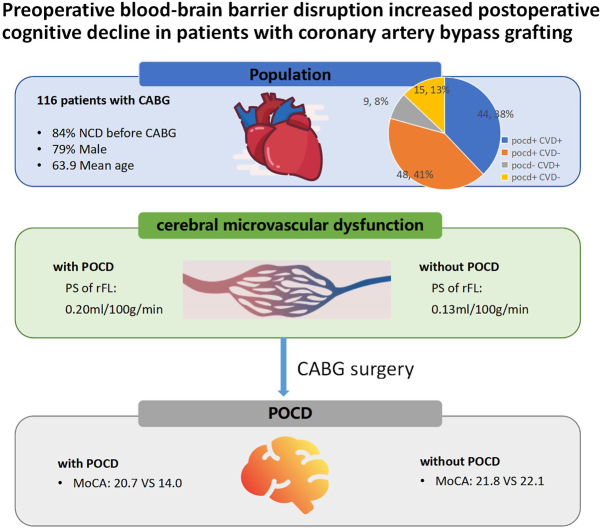
Structured graphical abstract. Preoperative blood-brain barrier disruption increased postoperative cognitive decline in patients with coronary artery bypass grafting.

In this prospective study, CTP was employed to assess BBB function before CABG surgery, aiming to uncover the correlation between baseline BBB condition and POCD. This marks the first integration of neurostructural imaging with functional assessment to investigate neurocognitive changes in coronary heart disease patients following CABG. This study found that an increase in PS is associated with POCD. PS is currently one of the potential imaging biomarkers for reflect BBB disruption in clinical practice, indicating that BBB disruption might contribute to the development of POCD. In the future, additional research into the underlying mechanisms could be undertaken to elucidate the causes of POCD (Fig. 6).

### CAD and cognitive decline

CAD is a widespread cardiovascular condition that significantly impacts global populations. A meta-analysis indicated that CAD is associated with a 26% increased risk of future dementia^[10]^. CAD is linked to accelerated cognitive decline following the acute coronary syndrome^[11]^, with affected individuals also experiencing faster decline over 5 years^[12]^. Factors contributing to cognitive deterioration post-acute coronary syndrome include older age, multiple myocardial infarctions, multiple cardiovascular risk factors of cardiovascular risk factors, emerging or worsening heart failure, rehospitalization, limited education, and decreased mobility^[13,14]^.

Although the exact mechanisms by which CAD induces long-term cognitive decline are not fully understood, several hypotheses have been proposed. For instance, CAD could directly affect cognitive functions by causing cerebral hypoxia and silent brain^[15]^ injuries. Postmortem studies have shown a correlation between ischemic heart disease and cerebral microinfarcts, suggesting an association between CAD and cerebral small vessel disease in the brain, which contributes to cognitive impairment. Additionally, shared vascular risk factors between CAD and cognitive decline^[16,17]^ play a crucial role. Long-term exposure to these risk factors, especially from early in life, may lead to cognitive deterioration through various biological pathways such as oxidative stress, immune responses, and endothelial dysfunction^[18]^. Imaging studies have further shown that vascular risk factors might contribute to neurodegenerative changes, including beta-amyloid deposition in the brain^[19]^. The interaction of these pathophysiological mechanisms can exacerbate both atherosclerotic and neurodegenerative lesions, leading to cognitive disturbances and dementia symptoms.

Our research has identified similar findings regarding cognitive decline. In our study, the average baseline MOCA score was 21 points, highlighting diminished cognitive function. Among those with preoperative MOCA scores under 26, 89.61% exhibited cognitive decline, increasing to 100.00% in patients with postoperative cognitive dysfunction (POCD). The higher incidence in our study may be attributed to all participants having severe CAD, along with multiple atherosclerosis risk factors such as previous myocardial infarction (20.69%), diabetes (49.14%), hypertension (68.10%), and dyslipidemia (68.10%).

### The mechanism of POCD

The study reveals that more than a quarter of CAD patients suffer cognitive decline following CABG surgery, possibly linked to preoperative disruptions in the BBB^[4,20,21]^. POCD is prevalent after cardiac surgery, significantly affecting both acute and long-term clinical outcomes and patient quality of life^[22]^. Research shows that approximately 29%^[23]^–53%^[2]^ of cardiac surgery patients develop measurable POCD soon after surgery. Our findings corroborate these figures, with 20.68% of CAD patients experiencing POCD post-CABG surgery, aligning with prior research.

Although POCD is a common neurological complication following CABG, the exact causes remain unclear. Current research suggests potential mechanisms such as microembolism during surgery or cerebral hypoperfusion^[24,25]^ due to hypotension during cardiopulmonary bypass^[26,27]^. Despite patients undergoing valve surgeries facing a higher rate of microemboli, and significantly more macroemboli compared to those undergoing CABG, no clear link was found between these emboli and the occurrence of POCD postoperatively^[24]^. A study using transcranial Doppler to monitor middle cerebral artery blood flow velocity during anesthesia induction and cardiopulmonary bypass indicated that reduced blood flow at any stage could lead to POCD, suggesting that diminished cardiac output might exacerbate neurological damage^[25]^. A prolonged reduction in blood flow can potentially cause ischemic brain injury. The exact duration of reduced blood flow necessary to induce neurocognitive disorders continues to be a key area of research. Additionally, BBB disruption is another suspected mechanism for POCD.

### BBB disruption and POCD

Cardiopulmonary bypass may cause BBB disruption, which significantly correlates with cognitive decline levels^[4]^. Merino JG’s study indicated that about half of the cardiac surgery patients showed BBB disruption^[3]^ through magnetic resonance (MR) imaging, particularly within the first 24 hours post-surgery. While the precise mechanism behind this link is still not fully understood, it is believed that postsurgical systemic inflammation leading to cerebral inflammation could be a major factor. Analysis of cerebrospinal fluid from cardiac surgery patients shows changes in the fluid’s albumin ratio, indicating BBB disruption. Elevated levels of IL-6 and IL-8 in the spinal fluid suggest cerebral inflammation, while increased S-100B and glial fibrillary acidic protein levels indicate glial cell damage^[20]^. Lin *et al*^[28]^ reported using standard CTP data to map the permeability surface, which could prompt BBB destruction. Some other studies have also used PS to observe BBB damage and related clinical outcomes in clinical practice^[29–32]^.

This study measured the PS of the frontal and temporal lobes before CABG in 116 patients. The findings indicated that PS was higher in patients with POCD than in those without, particularly in the frontal lobe. The model demonstrated consistent predictive effects across various subgroups, regardless of age, gender, diabetes mellitus history, hypertension history, left ventricular ejection fraction, and cardiovascular status. Elevated preoperative PS is associate with the occurrence of POCD after CABG.

### Executive function of the frontal lobe after POCD

While both the temporal and frontal lobes are supplied by the internal carotid artery, this study found that POCD primarily impairs the executive function of the frontal lobe. CTP imaging analysis suggests that the frontal lobe’s BBB is the most severely affected.

A rigorous cohort study confirmed a significant link between preoperative BBB damage in the frontal lobe and the development of POCD^[4]^, supporting earlier observational studies that found widespread BBB disruption in these patients, particularly affecting the frontal lobes^[4]^. Although executive dysfunction often results from frontal lobe injury, it generally arises from distributed processes that support executive functions. The prefrontal cortex includes the lateral prefrontal, orbitofrontal, medial prefrontal, and frontopolar regions, each associated with distinct cognitive and behavioral syndromes. The frontopolar cortex plays a critical role in multitasking and prospective memory, involving maintaining primary goals while managing or planning other tasks^[33]^.

In this study, patients with POCD showed a significant decline in postoperative orientation, a key function of the frontal lobe.

### POCD and outcome of CABG

The research also established a correlation between POCD and an extended duration of ICU stay. POCD has long-term implications for patient health and quality of life, including prolonged hospital stays^[34]^. Although the majority of patients with POCD show a gradual normalization of cognitive functions, with a significant decrease from approximately 40% on the fourth postoperative day to 2.5% at the 3-month follow-up^[26]^, acute POCD is strongly linked to long-term cognitive decline 6 years after surgical intervention^[2,35]^. This suggests that while most patients recover, those with persistent POCD face severe long-term effects.

A major concern is that prolonged POCD may increase the risk of developing dementia years after the surgery. A comprehensive study covering all Swedish patients who underwent CABG over 23 years explored the long-term risk of dementia onset compared to a similarly matched control group. The findings indicated that CABG patients under 75 years old showed an increased risk of developing dementia, while those over 75 years had a reduced risk compared to their age-matched controls^[36]^.

### Study strengths and limitations

To the best of our knowledge, this is the first prospective cohort study that employs quantitative neuroimaging to specifically evaluate the relationship between POCD after CABG and BBB function. To enhance the quality of evidence and minimize potential biases, our study was blinded at multiple levels: Cardiac surgeons and radiology investigators were unaware of the neurocognitive evaluations and the MoCA, while neurology and radiology investigators were not informed about the cardiac surgeries. Additionally, patients were blinded to both cardiac and cerebral assessments.

The present study has several limitations: First, it is a single-center, observational study with a relatively small sample size. Second, there was significant attrition due to poor coordination between neuroimaging and scale examination, compounded by postoperative physical weakness. Third, the direct relationship between PS and BBB damage still requires some mechanistic studies to prove the direct link between the two. Due to the limitations of clinical methods for BBB injury and ethical requirements, this study was unable to obtain the gold standard for BBB injury (cerebrospinal fluid/serum albumin ratio). In the future, we hope to conduct animal research to make up for this deficiency. POCD only reflects the early postoperative period and lacks long-term follow-up data. All patients underwent general anesthesia and were administered specific anesthetic drugs, so we cannot rule out the potential negative impact of these drugs on cognitive function. Although our analyses strongly suggest an association between BBB disruption and POCD, the potential for multiple statistical testing and the risk of a Type I error cannot be entirely dismissed. Finally, the results should be considered hypothesis-generating rather than definitive. Despite these limitations, the blinded and prospective nature of this study, along with comprehensive neurological evaluations and the use of multiple diagnostic techniques, strongly support our results.

## Conclusions

Almost one-fifth of patients develop POCD following CABG surgery, and preoperative elevation of frontal lobe PS is associated with POCD. These findings suggest that CTP-PS may serve as an imaging biomarker to identify patients at high risk for POCD.

## Data Availability

The original contributions presented in the study are included in the article/supplementary material, further inquiries can be directed to the corresponding author/s.

## Research registration unique identifying number (UIN)

https://www.clinicaltrials.gov/, NCT06275139.

## Clinical competencies

POCD frequently occurs following CABG surgery, significantly affecting both short-term and long-term patient outcomes and overall quality of life. Currently, predicting which patients will develop POCD prior to surgery is challenging. Despite numerous explored risk factors, predictive capabilities remain limited due to the absence of a quantifiable marker. This study introduces the use of a CTP index to quantitatively assess vulnerable brain tissue in the preoperative phase. By linking potential structural mechanisms of POCD development with clinical outcomes, this approach presents a novel method for identifying patients at risk for POCD preoperatively. It also suggests that future research could use neuroimaging techniques to more accurately identify at-risk patients, setting the stage for preoperative intervention studies aimed at preventing the condition.

## Central message

Preoperative elevation of PS may be due to BBB impairment, which is associated with POCD. This study introduces an imaging technique for early identification of risk for POCD before CABG surgery.

## Perspective statement

Disruptions in the preoperative blood-brain barrier (BBB) are associated with POCD in patients with CABG. Patients with CAD exhibit a higher susceptibility to cognitive decline. The preoperative elevation of PS may be due to BBB impairment, which is associated with POCD following CABG surgery.
